# Real‐Space Imaging of Intrinsic Symmetry‐Breaking Spin Textures in a Kagome Lattice

**DOI:** 10.1002/advs.202404088

**Published:** 2024-08-19

**Authors:** Caihong Xie, Yongcheng Deng, Dong Zhang, Junbo Li, Yimin Xiong, Mangyuan Ma, Fusheng Ma, Wei Tong, Jihao Wang, Wenjie Meng, Yubin Hou, Yuyan Han, Qiyuan Feng, Qingyou Lu

**Affiliations:** ^1^ University of Science and Technology of China Hefei 230026 China; ^2^ Anhui Province Key Laboratory of Low‐Energy Quantum Materials and Devices High Magnetic Field Laboratory HFIPS Chinese Academy of Sciences Hefei 230031 China; ^3^ State Key Laboratory for Superlattices and Microstructures Institute of Semiconductors Chinese Academy of Sciences Beijing 100083 China; ^4^ Department of Physics School of Physics and Optoelectronics Engineering Anhui University Hefei 230039 China; ^5^ Hefei National Laboratory Hefei 230094 China; ^6^ Anhui Provincial Key Laboratory of Magnetic Functional Materials and Devices Anhui University Hefei 230039 China; ^7^ School of Physics and Technology Nanjing Normal University Nanjing 210046 China

**Keywords:** Fe3Sn2, kagome magnets, Magnetic force microscopy, phase transition, spin reorientation

## Abstract

The electronic orders in kagome materials have emerged as a fertile platform for studying exotic quantum states, and their intertwining with the unique kagome lattice geometry remains elusive. While various unconventional charge orders with broken symmetry is observed, the influence of kagome symmetry on magnetic order has so far not been directly observed. Here, using a high‐resolution magnetic force microscopy, it is, for the first time, observed a new lattice form of noncollinear spin textures in the kagome ferromagnet in zero magnetic field. Under the influence of the sixfold rotational symmetry of the kagome lattice, the spin textures are hexagonal in shape and can further form a honeycomb lattice structure. Subsequent thermal cycling measurements reveal that these spin textures transform into a non‐uniform in‐plane ferromagnetic ground state at low temperatures and can fully rebuild at elevated temperatures, showing a strong second‐order phase transition feature. Moreover, some out‐of‐plane magnetic moments persist at low temperatures, supporting the Kane–Mele scenario in explaining the emergence of the Dirac gap. The observations establish that the electronic properties, including both charge and spin orders, are strongly coupled with the kagome lattices.

## Graphical abstract

Using ultra‐high‐resolution magnetic force microscopy, a novel type of periodic and regular hexagonal spin texture is revealed in the frustrated kagome Fe_3_Sn_2_ magnet. The results suggest that the kagome lattice can strongly modulate the magnetic order over a much larger scale and with more regular symmetries than previously thought.

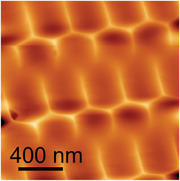

## 1 Introduction

The kagome materials, characterized by their unique corner‐sharing triangular lattice structure, have attracted much attention for the discovering of various exotic quantum phases.^[^ ^1^, ^2^, ^3^, ^4^, ^5^, ^6^, ^7^, ^8^, ^9^ ^]^ Theoretical and experimental endeavors in this field range from exploring spin‐liquid phases in kagome antiferromagnets,^[^ ^4^ ^]^ to studying topological matter, and most recently, investigating unconventional superconductivity.^[^ ^5^ ^]^ These phenomena are mainly induced by the complex intertwining of the unusual electronic orders with the kagome lattice and the underlying crystal symmetry. Of particular interest are the variety charge orders, such as the symmetry‐broken charge density waves (CDWs), revealed in both the non‐magnetic and magnetic kagome materials.^[^ ^5^, ^6^, ^7^, ^8^, ^9^ ^]^ For kagome magnets, various magnetic ground states have been recognized during the past decade, such as the noncollinear 120° spin order in antiferromagnet Mn_3_Sn^[^ ^10^, ^11^ ^]^ and the lattice‐induced flat‐band ferromagnetism in Fe_3_Sn_2_.^[^ ^12^, ^13^, ^14^ ^]^ However, the intrinsic spin orders, particularly their interplay with the kagome lattice, has never been directly observed to date, despite its essential role in comprehending the nontrivial electronic ground states dominated by magnetism in a kagome lattice.

To explore the interplay between magnetism and crystalline structure, ferromagnetic (FM) kagome metals are promising candidates, as the relatively strong magnetic signal facilitates both imaging and transport measurements. Although recent studies have primarily focused on the unusual transport properties and correlated non‐trivial spin textures under finite fields,^[^ ^1^, ^15^, ^16^, ^17^, ^18^, ^19^, ^20^, ^21^, ^22^, ^23^ ^]^ indirect evidence has indicated that the magnetic configurations in the FM material Fe_3_Sn_2_ can be significantly impacted by its peculiar lattice geometry, inspiring us to visualize the intrinsic spin state under the influence of the underlying symmetry properties.^[^ ^1^, ^2^, ^12^, ^24^ ^]^

In this work, we study the spin structures of Fe_3_Sn_2_ at the nanoscale from 300 K to low temperatures at zero field by using a home‐made high‐resolution scanning magnetic microscopy (MFM).^[^ ^25^ ^]^ We observe an unexpected honeycomb lattice form of spin textures in Fe_3_Sn_2_ in zero field. Compared with the kagome lattice, its sixfold rotational symmetry is broken due to the competition between the tilted uniaxial magnetic anisotropy and the kagome symmetry. It transforms into the in‐plane magnetic ground state at low temperatures, not the spin glass state as previously reported.^[^ ^15^, ^19^, ^20^, ^26^ ^]^ In addition, MFM results provide strong evidence that the temperature‐dependent phase transition in Fe_3_Sn_2_ is a second‐order phase transition, which would be significant in understanding the temperature‐induced change in the band structure when considering spin orientations. These results identify the intrinsic nature of the coupling between geometry, spin, charge and correlation in kagome magnets.

## 2 Results

### 2.1 Spin Reorientation in Fe_3_Sn_2_

**Figure 1:**
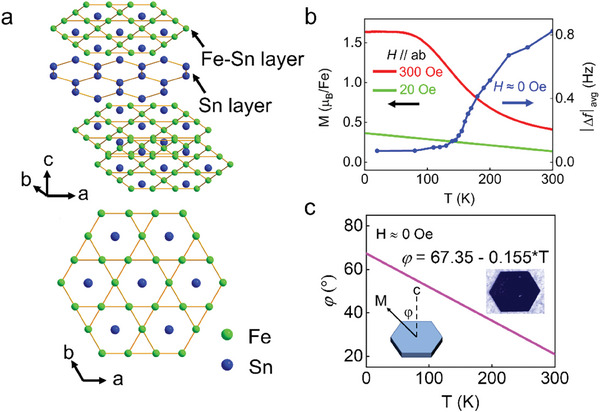
Crystal structure and spin‐reorientation transition in Fe_3_Sn_2_. a) Top panel: Side view of the structure of Fe_3_Sn_2_, showing the alternative stacking of FeSn kagome bilayers and Sn layers along the *c*‐axis. Lower panel: top view of the Fe‐Sn layer. b) Temperature‐dependent magnetization curves of Fe_3_Sn_2_ along *ab*‐plane (red and cyan lines) and *c*‐axis (M*_c_*_‐axis_∝ | Δ *f* |_avg_^[^ ^41^ ^]^; bule line). The red (cyan) line is recorded by a magnetometry in an applied field of 300 Oe (20 Oe), between 2 and 300 K. While the blue line is obtained from the MFM images during ZFC. c) The temperature dependence of the angle between the M (magnetic easy axis) and the *c*‐axis. The linear equation in the inset, derived from the cyan curve in Figure 1b, expresses the variation of the angle with temperature. The insert on the right shows an Fe_3_Sn_2_ single crystal. It grows in a hexagonal shape with a thickness typically less than 100 µm but can with a large lateral size of the order of millimeter.

As shown in **Figure** **1**a, Fe_3_Sn_2_ has a layered crystal structure composed of a Sn spacing layer sandwiched between kagome FeSn bilayers. It crystallizes in the space group $R\bar{3}m$ with hexagonal lattice constants *a* = 5.3 and *c* = 19.8 Å. Consequently, Fe_3_Sn_2_ can crystallize in a rather regular hexagonal shape, as shown in the insert of Figure 1c, demonstrating the high quality of the sample. Fe_3_Sn_2_ is a soft ferromagnet (Figure S1, Supporting Information) with a high Curie temperature of ≈ 640 K^[^ ^27^, ^28^, ^29^ ^]^ and its magnetic response mainly arises from the Fe atoms. The magnetic moments are largely along *c*‐axis at high temperatures but gradually reorient to kagome plane (*ab*‐plane) as the temperature decreases, coined as spin reorientations.^[^ ^13^, ^17^, ^22^, ^30^, ^31^ ^]^ Bulk magnetization data on cooling along the *ab*‐plane with a small field of 300 Oe is shown in Figure 1b (red line). The increase in magnetization from 300 K to 80 K is attributed to the reorientation of the spins, and the plateau below 80 K is suggested to be the formation of spin glass ground state at low temperatures.^[^ ^15^, ^19^, ^20^ ^]^

### 2.2 The Natural Fine Magnetic Structure in Zero Magnetic Field

**Figure 2:**
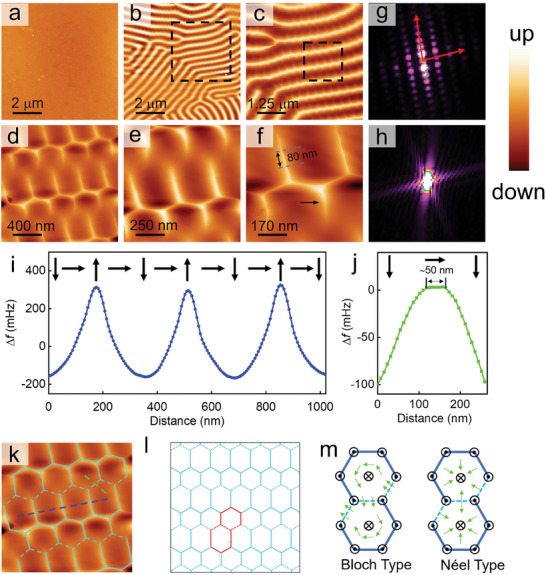
Magnetic structure at room temperature. a) Topography. The color scale is 10 nm. b–f) MFM images taken at the same location of the sample but with the scan size gradually decrease from 10 to 0.7 µm. The color scales are 6.4, 5.2, 2.3, 1.1, and 1.1 Hz, respectively. Note that the color scales are not normalized for all the MFM images in this text. (g), (h) are FFT of (c), (f). (i) and (j) are line profiles along the blue and cyan dashed lines in (k), respectively. The black arrows in the top of the images show how the direction of moments vary. k) Duplication of (d) to illustrate the spin structure. l) Outlines of the hexagonal lattice structure. In (k) and (l), solid and dashed lines representing out‐of‐plane and in‐plane magnetic moments, respectively. One repeat unit is marked by red color. m) Schematic of possible spin configurations for the repeat units based on Bloch and Néel models. The cyan arrows indicate the direction of the in‐plane magnetizations.

To study the intrinsic magnetic structures of Fe_3_Sn_2_, all the MFM images in this paper were measured in zero external magnetic field (see more details in the “MFM measurements and data analysis” section in Methods). The crystal is cleaved under ambient condition and then quickly inserted into the MFM head.^[^ ^25^, ^32^ ^]^ The topographic image and the corresponding MFM image at the same region are presented in **Figure** **2**a,b, respectively. No direct correlation is found between them. Figure 2b, collected at a relatively large scan size of 10 µm, shows an alternating bright and dark strip pattern. It is classified as out‐of‐plane FM domains with magnetization directions either parallel or anti‐parallel to the *c*‐axis, confirmed by both Lorentz transmission electron microscopy (LTEM) and other MFM results.^[^ ^12^, ^15^, ^16^, ^30^, ^33^ ^]^ Here, to uncover the intrinsic spin configurations, a series of zoom‐in MFM measurements was performed sequentially (Figures. 2c–f). For instance, Figure 2c was recorded at the boxed region of Figure 2b, and the enlarged image of the boxed region in Figure 2c produced Figure 2d. The other boxes are omitted for simplicity. Unexpectedly, we discover an entirely different spin pattern under a higher spatial resolution (Figure 2d). It shows a rather regular shape with straight boundaries, and the angles between adjacent boundaries are approximately the same (≈120°). Further enlarged MFM images, as displayed in Figure 2e,f, reveal additional details of these spin textures. They also confirm that the observed magnetic signal is a result of spin ordering, not periodic electrical noise, as the latter does not change as the scan size decreases. Besides, these spin textures show a relatively large contrast of the order of 1 Hz, while the noise signal is typically less than 20 mHz in our MFM,^[^ ^10^, ^32^ ^]^ further excluding the influence of noise. Therefore, this MFM contrasts should be assigned to the true spin textures, and the original stripe‐like MFM contrast in Figure 2b, however, should be caused by the average effect of adjacent spin textures.

**Figure 3:**
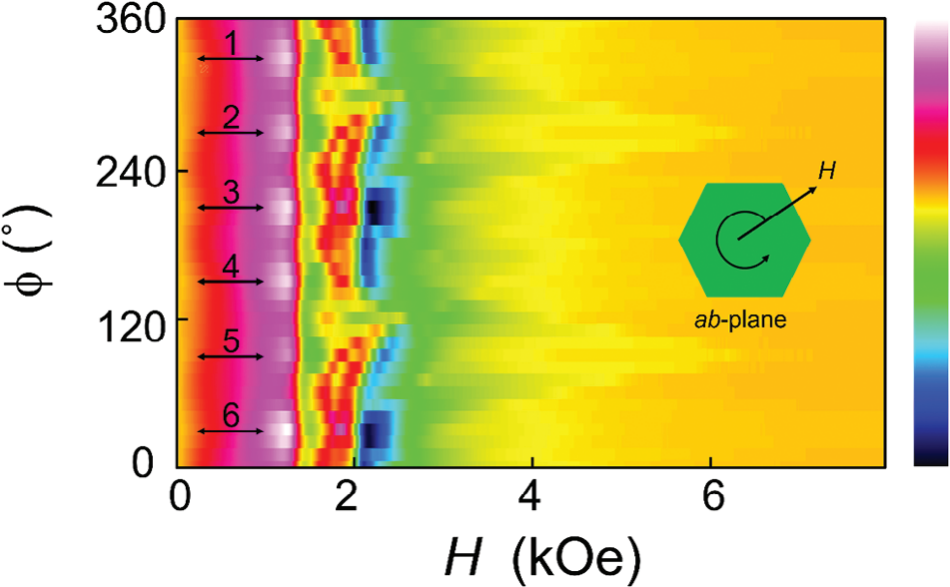
The ESR measurements with the magnetic fields applied in the *ab*‐plane at 300 K. Please note that the initial orientation of the magnetic fields does not align with a specific axis of the sample. The six peaks in the ESR spectra are marked by the numbers 1 to 6. The inset shows the direction of the applied field with respect to the sample.

It is noted that the identified fine magnetic structures in Figure 2d,e shows a honeycomb‐like pattern but with some edges periodically missing. To represent its magnetic structure more clearly, solid and dashed black lines are drawn along these two different categories of edges in Figure 2d, yielding Figure 2k. Two distinct hexagonal cells, measuring 300 nm and 180 nm respectively, have been identified. These dimensions are comparable to the size of the bubbles or skyrmion bubbles in this material,^[^ ^15^, ^16^, ^17^, ^27^ ^]^ indicating that it is a new type of spin texture. In addition, we show a schematic representation of the lattice form of these spin textures, as depicted in Figure 2l, where a repeating unit cell is highlighted in red. To further study the periodicity and the symmetry of this hexagonal spin texture (HST), we perform 2D fast Fourier transforms (FFTs) of Figure 2c,f, and the results are shown in Figure 2g,h, respectively. In Figure 2g, the FFT is very similar to the electron diffraction pattern of a single crystal, suggesting the existing of translation symmetry as marked by the red arrows, and is fully consistent with the experimental results and schematic provided in Figure 2d,l. The FFT pattern of several spin textures additionally confirms that these spin textures indeed possess a hexagonal shape (Figure 2h) and are closely linked to the sixfold rotational symmetry of the kagome lattice. Besides, electron spin resonance (ESR) measurements (Bruker EMX plus 10/12) were performed to further illustrate the correlations between the magnetic spins and the symmetry of the lattice, as shown in **Figure** **3**. The magnetic field was applied within the *ab*‐plane and measured through a 360° rotation, as illustrated in the schematic diagram in Figure 3. In the low field range (less than 2000 Oe), the ESR spectra show six peaks with nearly identical positions and strength. The angle difference between adjacent peaks is 60°, indicating a strong correlation between the magnetic moments and the sixfold rotational symmetry of the kagome lattice.

In addition, MFM results of other four samples are showed in Figure S2 (Supporting Information). Both samples 1 and 3 show dendritic domains, which are completely different from the periodic hexagonal spin textures presented in the main text. Note that sample 3 shows smaller branched structures. Samples 2 and 4 show strip magnetic structures with smooth boundaries. Although the stripes are clearly separated and periodic, hexagonal structures are not found, as clearly revealed by the zoomed‐in image shown in Figure S2e (Supporting Information). One should note that at this scan scale, the hexagonal magnetic structures can be clearly revealed (Figure 2). Therefore, we suggest that the strip domains have limited impact on the formation of the hexagons. We infer that the formation of the hexagonal spin textures is strongly dependent on the quality of the samples, in other words, the lattice structures. These features strongly suggest that the intrinsic spin order is statically modulated by the periodical kagome lattice.

To reconstruct this hexagonal spin configuration, line profiles across these two different kinds of edges are analyzed (Figure 2i,j). Although MFM imaging is only sensitive to the out‐of‐plane magnetizations and is incapable of distinguishing in‐plane spin directions, useful information can still be acquired, such as an entirely zero signal indicating a fully in‐plane magnetic state. Both line profiles show the noncollinear nature of the magnetic moments in Fe_3_Sn_2_. For solid edges, the spins point up and swirl down at the core regions. However, for the dashed edges, a smooth zero‐signal platform indicates that spins are almost in‐plane at the perimeter area and also continuously rotate in the downward direction at the hexagon centers. The width of the platform can extend up to 50 nm, providing strong evidence that a boundary does exist at this location, even though it is invisible in the MFM images (more results and discussion can be found in Figure S3, Supporting Information). On this basis, two possible spin configurations based on Bloch type or Néel type are proposed in Figure 2m. In Figure 2k,l, one can note that the repeating unit cell consists of two non‐identical hexagons: a regular one and an elongated one. From this perspective, the Bloch type seems more likely to be correct, for the helicity of the two hexagons is different: the rotational direction of the in‐plane moments around the core is clockwise and counterclockwise, respectively.^[^ ^34^ ^]^ In contrast, the Néel type is composed of two identical spin textures.

After the establishment of the HST, greater attention is now being directed toward the inner magnetic structure of the hexagon. As shown in Figure 2f, the highest resolution image, the short boundaries (bright lines of the small hexagon) always bend to the left, as indicated by the black arrow. These characteristics strongly suggest that they are indeed true magnetic structures. More interestingly, we find that long boundaries (bright lines of the large hexagon) display wave‐like structures with a periodicity of ≈80 nm. These can be attributed to zigzag magnetic domain walls, which have been widely reported. The difference between the short and long boundaries mentioned above should be that the short boundaries are too short to form the periodic structures. We further propose that these magnetic structures form in order to minimize the boundary energy.

Additionally, as shown in Figure 2b,c, which were acquired over relatively large scanning areas, it should be noted that some magnetic transition regions exist. Analyzing these magnetic transition regions is essential for understanding the formation of HSTs. For example, in Figure 2c, the third line from the top bifurcates near the label “c”, resulting in two very narrow lines at the point of divergence (The enlarged MFM image is included as Figure S4, Supporting Information). The two lines are very narrow, indicating a sharp magnetic flip transition. This is further corroborated by the very sharp line profile signal across the boundary. Furthermore, it can be observed that hexagonal spin textures are disrupted in this region, likely due to the effect of spatial confinement. The magnetic moments in this region also exhibit a non‐linear nature, with the moments flipping continuously. Besides, one should note that the direction of the hexagons changes with the orientation of the main lines. The change in the orientation of the hexagons in the left bottom corner of Figure 2c is depicted in Figure S5 (Supporting Information). In this region, the main lines, marked by solid red lines, have an angle difference of ≈45°. It is observed that the direction of the hexagons is always perpendicular to these main lines.

**Figure 4:**
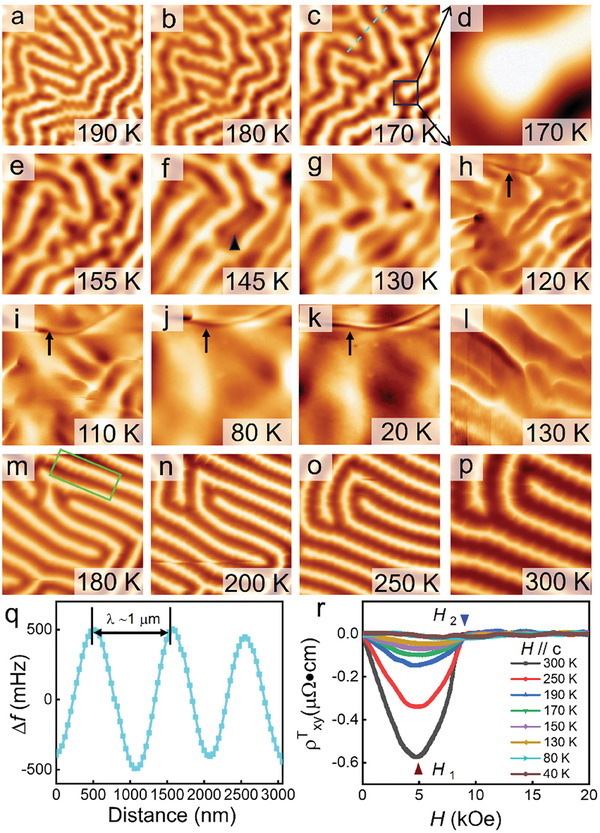
MFM images during thermal cycling. a–p) Selected MFM images taken at the same area in zero field during thermal cycling from 300 to 20 K and then back to 300 K again. d) Magnetic structure obtained by magnifying the boxed region in (c). The color scales are 3.33, 3.32, 2.02, 1.41, 1.44, 1.03, 0.81, 0.70, 0.60, 0.57, 0.56, 1.87, 3.86, 5.89, 6.45 and 6.71 Hz, respectively. The scanned areas are 9.0 × 9.0, 8.7 × 8.7, 8.0 × 8.0, 1.4 × 1.4, 8.0 × 8.0, 8.0 × 8.0, 8.0 × 8.0, 8.0 × 8.0, 8.0 × 8.0, 8.0 × 8.0, 8.0 × 8.0, 7.0 × 7.0, 6.7 × 6.7, 6.2 × 6.2, 5.3 × 5.3 and 3.5 × 3.5 µm^2^, respectively. q) Line profile along taken along the dashed cyan line in (c). r) $\rho_{xy}^{T}$ versus *H* for Fe_3_Sn_2_ single crystal at different temperatures.

The resolution of the definitive spin configuration is beyond the capacity of the MFM. A spin‐sensitive microscope with higher resolution like LTEM may be a useful tool to solve this problem, but the samples have to be thinned down to a thickness typically less than 200 nm first. At this scale, the nano size effect or spatially geometric confinement is nonnegligible and can destroy this intrinsic spin structure.^[^ ^15^, ^16^, ^17^, ^18^ ^]^ Although various non‐trivial spin textures have been successfully resolved in this material family, this HST has not been captured until now. It is noteworthy that this HST is very similar to the hexagonal skyrmion lattice in crystal lattices with tetragonal or hexagonal structure,^[^ ^35^ ^]^ but it is non‐topological, as indicated by both its broken spin structures and the zero topological Hall resistivity at zero field (**Figure** **4**r). More interestingly, its rotational symmetry is broken compared to the sixfold symmetry of the kagome lattice, even though its spin ordering is formed by this peculiar lattice geometry.^[^ ^12^ ^]^

### 2.3 Evolution of the Magnetic Structures on Thermal Cycling

After discerning this intricate magnetic structure, we studied its temperature dependence under a zero magnetic field to ascertain its formation mechanism and properties, as illustrated in Figure 4 (the complete set of images can be found in Figure S6, Supporting Information). All the MFM images were acquired in the same region but with varied scan sizes. After zero‐field cooling (ZFC) to 190 K, strips with perpendicularly aligned protrusions are formed. These protrusions are periodicity arranged, which can be considered as residues of the HST lattice. At 170 K, these protrusions disappear, and stripe domains with smooth boundaries are formed. These strips also exhibit a noncollinear nature with a periodicity of ≈1 µm, as shown in Figure 4q and Figure S7 (Supporting Information). As we continue this ZFC process, the strips begin to broke, as shown in Figure 4c, with close‐ups depicted in Figure 4d. The termination of the strip exhibits a circular shape, showing a spin configuration much like a half‐skyrmion.^[^ ^18^, ^36^ ^]^ Dramatic changes occur at 145 K: the boundaries between strips become indistinct (marked by a small arrowhead) and finally merge together at 130 K, due to the gradual rotation of the spin direction to the *ab* plane. We note that a narrow black line appears at the left top of image at 120 K, as indicated by the black arrow, and it elongates and becomes more evident at 110 K. Consider that the magnetic moments are almost in‐plane at low temperatures, this line should be ascribed to the domain wall at low temperatures. A line profile across this domain wall shows that it is Néel type domain wall (see Figure S8, Supporting Information).^[^ ^37^ ^]^ However, strong contrasts still exist in the regions enclosed by the domain wall, indicating that some amounts of moments are still out‐of‐plane (see Figure S9, Supporting Information). The images show little change below 80 K, in excellent agreement with the temperature‐dependent magnetization shown in Figure 1b. Therefore, the low‐temperature phase should be the in‐plane ferromagnetism.

It is noteworthy that significant non‐uniform magnetic contrast can still be found even in a single domain; this is in striking contrast to the 3*d* transition metal compounds, in which each domain is usually occupied by a single pure phase. This suggests that the spin ordering in kagome materials, even in their FM ground state, can still be significantly influenced by the geometrically frustrated lattice structure,^[^ ^29^ ^]^ although the magnetic anisotropy now plays a more prominent role. On the other hand, our mapping reveals that a considerable number of FM moments remains aligned out‐of‐plane at low temperatures (Figure 4k; Figure S9, Supporting Information). This observation provides additional support for the Kane–Mele scenario in explaining the formation of the Dirac gap in Fe_3_Sn_2_ at low temperatures.^[^ ^1^ ^]^ At high temperatures, the spontaneous magnetization is out‐of‐plane, and a Kane–Mele spin–orbit‐type interaction produces the Dirac gap. At low temperatures, it has been proposed that the spontaneous magnetization is in‐plane, causing the failure of the Kane–Mele model. However, our observation suggests that a substantial number of out‐of‐plane magnetized moments still persist, potentially facilitating the splitting of Dirac crossing and the generation of a Dirac gap. Note that the low‐temperature out‐of‐plane magnetization of Fe_3_Sn_2_ samples is challenging to detect through transport means due to their nature as soft magnetic materials, exhibiting a zero macroscopic magnetization. Nevertheless, it can be unambiguously detected by MFM, regardless of whether the local magnetic moments are oriented upward or downward, as MFM is highly sensitive to out‐of‐plane signals.

It is found that the magnetic anisotropy, which can be gradually tuned by the temperature‐driven spin reorientation transition, can have a primary effect on the spin ordering as depicted by the ZFC MFM results.^[^ ^15^, ^17^, ^38^ ^]^ To further confirm this mechanism, zero‐field warming (ZFW) MFM measurements were subsequently applied. As the temperature is elevated to 130 K, domain wall disappears, but narrow lines develop and can expend to form periodical strips at 180 K (Figure 4m). Protrusions begin to develop, as marked by the green rectangle, and behave the same as in the ZFC process. As the temperature further increases, these protrusions prevail and serve as the edges of the hexagons, dividing the whole sample into a hexagonal spin‐lattice. The magnetic phase transition during thermal cycling is completely reversible, and the temperature at which the distinctive magnetic structure emerges aligns perfectly, irrespective of the temperature history. Moreover, the magnetization data extracted from our MFM images (blue line in Figure 1b, with the detailed methodology outlined in the “Experimental” section) align well with the bulk magnetization curve (red line in Figure 1b), consistently demonstrating a smooth spin transition. Therefore, we suggest that it may be a second‐order phase transition because the magnetic moments gradually rotate from out‐of‐plane to in‐plane and no new phase is found during this process, which is very distinct form the abruptly changes of the domains during a first‐order phase transition.^[^ ^39^ ^]^ This can be further confirmed by the temperature‐dependent magnetization measurements along the *ab*‐plane with a small applied field, as shown by the cyan line in Figure 1b. Interestingly, we find a linear increase in the magnetization along the *ab*‐plane. Based on this, the temperature dependence of the angle between the M (magnetic easy axis) and the *c*‐axis can be estimated, as illustrated in Figure 1c. We also note that this result aligns exceptionally well with that reported in ref. [15]. Thus, the variation in the portion of the moments along the *c*‐axis with changing temperatures can now be estimated (Figure S10, Supporting Information). We can clearly see that the magnetic moment is predominantly out‐of‐plane in the high‐temperature region. As the temperature decreases, the magnetic moment gradually transitions to the in‐plane direction.

Besides, the temperature dependence strongly suggests that the competition between the uniaxial magnetic anisotropy and the influence of the kagome lattice induced the formation of this lattice from of spin textures at room temperature. The FM moments in Fe_3_Sn_2_ are naturally in a noncollinear form and can be organized into swirling HST under the influence of the kagome lattice. However, the angle that magnetic anisotropy makes with respect to the *c*‐axis breaks the rotational symmetry, leaving a symmetry‐breaking spin texture (Figure S11, Supporting Information).

In the formation of HST, several key factors should not be ignored. The first regards the overall symmetry of the crystal. Although each kagome layer possesses sixfold rotational symmetry, the stacking of neighboring kagome layers reduces the rotational symmetry from sixfold to threefold. This effect can also account for the broken rotational symmetry of the HST. Although a similarly explanation has been widely adopted to describe the unusual symmetry‐breaking properties of CDWs in kagome lattices,^[^ ^9^ ^]^ it alone cannot explain our experimental results, as the observed HST lacks threefold rotational symmetry as well. For example, the reduction of rotational symmetry from the sixfold to twofold can produce a twofold charge order in KV_3_Sb_5_.^[^ ^40^ ^]^ One possibility is that the formation of this HST is a consequence of the synergetic effect, where the symmetry properties of the kagome lattice, uniaxial magnetic anisotropy, and different interlayer stackings all contribute to the formation this complex HST. The second factor concerns lattice distortion. Unlike the formation of unconventional CDWs, where the kagome lattice undergoes periodic distortion, the FeSn layer retains the pristine structure at 300 K.^[^ ^2^, ^12^ ^]^ Hence, the lattice distortion effect can be ruled out. It has been reported that the magnetic moments in Fe_3_Sn_2_ are induced by the exchange interaction within each hexagon of the kagome lattice and can in addition form long‐range FM order through the hexagons network.^[^ ^12^ ^]^ Compared with other spin orders, such as spin density waves, our results suggest that the kagome lattice can strongly modulate the magnetic order over a much larger scale and with more regular symmetries than previously believed.

### 2.4 Emergence of Topological Orders and Phase Diagram

**Figure 5:**
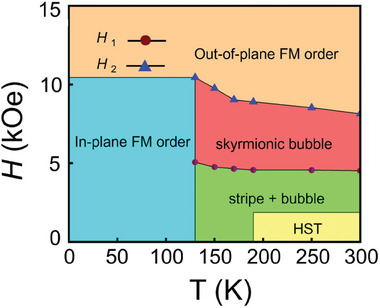
Magnetic phase diagram of Fe_3_Sn_2_ single crystal. HST is first found by MFM measurements. *H*_1_ and *H*_2_ correspond to the fields where the absolute value of $\rho_{xy}^{T}$ reach peak value and then vanishing, as shown in Figure 4r.

Although various topological orders in Fe_3_Sn_2_ above 130 K after an application of fields have been identified by previous studies,^[^ ^2^, ^15^, ^27^ ^]^ their correlations with the intrinsic magnetic states at zero field have rarely been revealed. Here, we extracted the topological Hall resistivities at several representative temperatures. In can be clearly found that, for temperatures below 130 K, strips disappear and the topological Hall resistivity is negligible over the entire field range. Recent studies have revealed the crucial role played by half‐skyrmions in the nucleation of skyrmion bubbles.^[^ ^18^ ^]^ As such, we suggest that these out‐of‐plane magnetized strips can be regarded as the precursor of the topological spin textures in Fe_3_Sn_2_. With the increasing field, these strips first break with a half‐skyrmion endpoint (Figure 4c,d) and then further nucleate into a skyrmion bubble. The topologically protected spin textures, however, seem not to be able to nucleate from the in‐plane FM state. By further combining the transport and the MFM measurements, a new phase diagram of Fe_3_Sn_2_ is drown in **Figure** **5**.^[^ ^15^, ^26^ ^]^ In our phase diagram, a honeycomb lattice is first revealed, and the low‐temperature phase is recognized as the in‐plane FM.

In summary, we presented a comprehensive high‐resolution MFM study on the intrinsic spin configurations in the strongly correlated kagome compound Fe_3_Sn_2_. We directly observed a new type of lattice form of spin textures in zero magnetic field and further present the first real‐space evidence for the coupling between the kagome lattice and magnetic order, which involves quantum interactions between spin, charge, lattice and flat‐band correlations, as well as symmetry‐broken and topological properties of spin ordering. Notably, these long‐period, regular spin‐lattice modulations are unexpected and absent in previous studies, revealing the versatile and exotic quantum physics of kagome magnets. Further experimental effort will focus on studying the spin textures at higher temperatures, where the magnetic anisotropy is mostly parallel to the *c*‐axis, and the natural rotational symmetry of the spin textures is expected to recover.

## 3 Experimental Section

### MFM Measurements and Data Analysis

All the MFM images showed in this text are collected with the MFM tip scanned along the kagome plane. The detailed structure of the home‐build MFM and the explanation of the image contrast can be found in ref.[25, 32, 42] A piezoresistive cantilever (PRC400; Seiko Inc.) is adopted as the force probe and its resonant frequency is ≈40 kHz and the shift in frequency (Δ*f*) is used for imaging. The tip of the cantilever was coated with 5 nm Ti, 50 nm Co, and then 5 nm Au as the protective layer. Before loading onto the MFM head, the tip was magnetized by using a permanent magnet with known magnetization direction, so the out‐of‐plane magnetization direction of the sample can be deducted. In this text, the dark (bright) contrast implying a downward (upward) magnetization direction. Though equipped with a superconducting magnet (Oxford Instruments), all images in Figure 2 were collected with the magnet removed to avoid the perturbation of residual magnetism produced by the steel parts of the magnet. Temperature‐dependent MFM measurements were performed with the MFM inserted into the magnet to get cryogenic environment. The field was tuned to zero but a small field can still exist, causing the corresponding quality of the images in Figure 4 is not as good as that in Figure 2. The upper limit of the residual magnetic field of our magnet is 300 Oe. Therefore, a true zero field background is urgently required for emergence of the delicate spin textures.

|Δ*f*|_avg_ data in Figure 1b are extracted from the MFM images at different temperatures. In our measurement, each image is, in fact, a 256 × 256 matrix. The corresponding |Δ*f*|_avg_ is the average of the absolute value of all elements in the matrix, as the sample is FM: the sign of the elements just represents the direction of the magnetic spin. The advantage of this method is that all pixels in an image are used (65536 pixels in all), which can largely reduce the random errors during measurements.

### Single‐Crystal Growth and Transport and Magnetization Characterization

Fe_3_Sn_2_ single crystals were synthesized by chemical vapor transport method with stoichiometric amounts of Fe (>99.9%; Alfa) and Sn (>99.9%; Alfa) powders.^[^ ^31^ ^]^ The corresponding magnetization and four probe transport measurements were performed using a SQUID magnetometer (Quantum Design) and a PPMS (Quantum Design), respectively. The $\rho_{xy}^{T}$ data showed in Figure 4r were extracted from the transverse Hall resistivity ρ_*xy*_ (Figure S12, Supporting Information) by subtracting the normal Hall resistivity $\rho_{xy}^{N}$ and the anomalous Hall resistivity $\rho_{xy}^{A}$: $\rho_{xy}^{T}=\;\rho_{xy}-\;\rho_{xy}^{N}-\;\rho_{xy}^{A}$, where the $\rho_{xy}^{N}=\;R_{N}H$ and $\rho_{xy}^{A}=\;R_{A}M$. The *R_N_*, *R_A_*, *H* and *M* denote normal Hall coefficient, cumulative anomalous Hall coefficient, out‐of‐plane field and magnetization, respectively. The *R_N_* is equal to the linear slope of the field dependence of the ρ_*xy*_ but with field larger than the saturation field. After subtracting the $\rho_{xy}^{N}$ parts from the ρ_*xy*_, the *R_A_* can be easily calculated by diving the $\rho_{xy}\;-\;\rho_{xy}^{N}$ by saturation magnetization in saturation field (Figure S1, Supporting Information), where the topological spin textures are annihilated and the corresponding $\rho_{xy}^{T}$ contribution is zero.

## Conflict of Interest

The authors declare no conflict of interest.

## Author Contributions

C.H.X. and Q.Y.F. performed the MFM measurements and characterized the materials, including the transport and magnetic measurements, under the guide of Q.Y.L.; J.B.L. synthesized the Fe_3_Sn_2_ single crystals in consultation with Y.M.X.; Q.Y.F. carried out the micromagnetic simulations with the help of M.Y.M and F.S.M; C.H.X., Q.Y.F. and Q.Y.L. performed the data analysis and wrote the paper; W.T. performed the ESR measurements. All authors participated in the discussion of the results. Q.Y.F. and Q.Y.L. supervised the project.

## Supporting information

Supporting Information

## Data Availability

The data that support the findings of this study are available from the corresponding author upon reasonable request.
