# Supplementary material for: Real‐Space Imaging of Intrinsic Symmetry‐Breaking Spin Textures in a Kagome Lattice
Source: Adv Sci (Weinh). 2024 Aug 19;11(39):2404088. doi: 10.1002/advs.202404088 (PMC11497059; doi:10.1002/advs.202404088)
Supplement: Supplementary file 1 — Supporting Information [file ADVS-11-2404088-s001.docx]

Supplementary Information for

**Real-space imaging of intrinsic symmetry-breaking spin textures in a kagome lattice**

Caihong Xie^1,2^, Yongcheng Deng^3^, Dong Zhang^3^, Junbo Li^2^, Yimin Xiong^4, 5, 6^ , Mangyuan Ma^7^, Fusheng Ma^7^, Wei Tong^2^, Jihao Wang^2^, Wenjie Meng^2^, Yubin Hou^2^, Yuyan Han^2^, Qiyuan Feng^2^*, Qingyou Lu^1,2^*

^1^ University of Science and Technology of China, Hefei 230026, China.

^2^Anhui Province Key Laboratory of Low-Energy Quantum Materials and Devices, High Magnetic Field Laboratory, HFIPS, Chinese Academy of Sciences, Hefei 230031, China.

^3^State Key Laboratory for Superlattices and Microstructures, Institute of Semiconductors, Chinese Academy of Sciences, Beijing 100083, China.

^4^Department of Physics, School of Physics and Optoelectronics Engineering, Anhui University, Hefei 230039, China.

^5^Hefei National Laboratory, Hefei 230094, China.

^6^Anhui Provincial Key Laboratory of Magnetic Functional Materials and Devices, Anhui University, Hefei 230039, China.

^7^School of Physics and Technology, Nanjing Normal University, Nanjing 210046, China.

Correspondence and requests for materials should be addressed to Q.Y.F. (email: fqyuan@hmfl.ac.cn) or to Q.Y.L. (email: qxl@ustc.edu.cn).


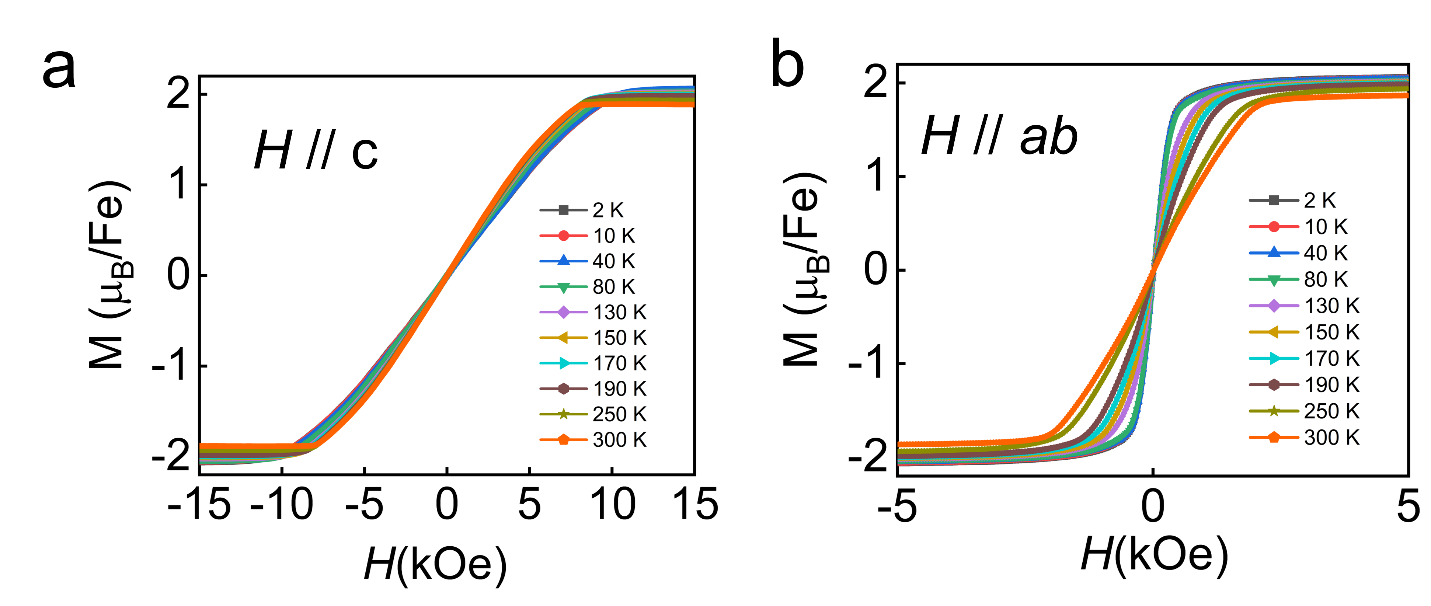


**Figure S1****.** Magnetization of bulk Fe_3_Sn_2_ for external fields along *c*-axis (**a**) or *ab* plane (**b**) at representing temperatures.


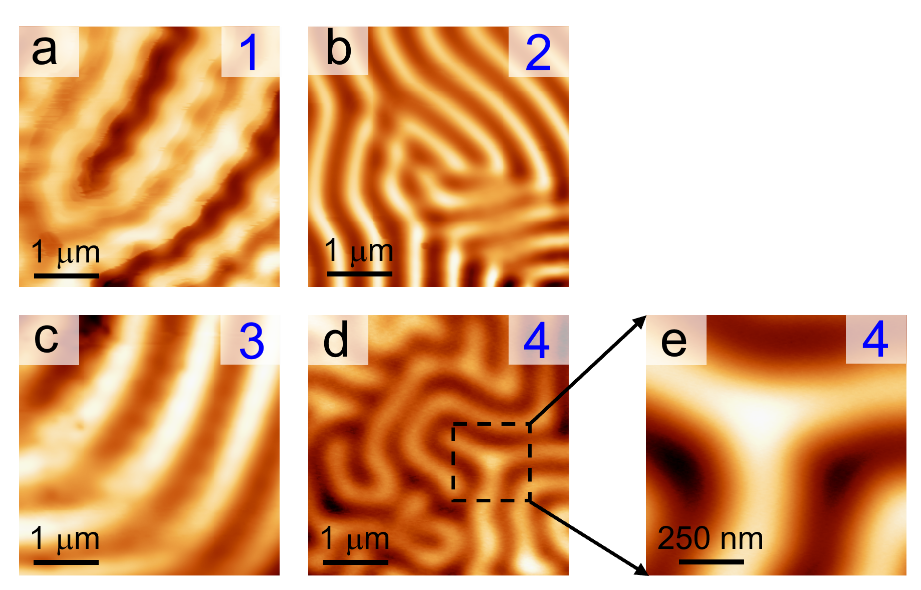


**Figure S2.** MFM images for the other four samples. (a)-(d), MFM images taken from 4 different samples. (e) The zoom-in image of the dashed squared region in (d).


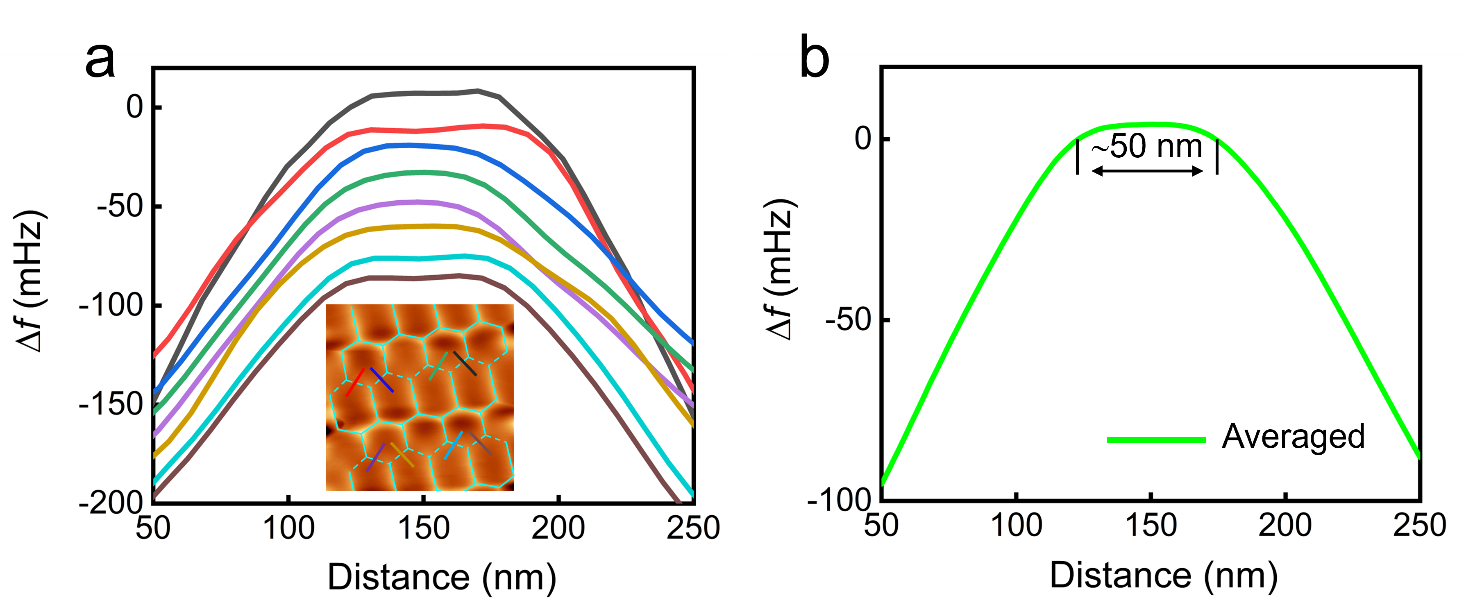


**Figure S3.** Statistical analyses of the MFM images. (a) Line profiles across eight dashed edges. The locations and orientations of these profiles are marked by the lines in the inset. (b) The averaged profile. The width of the dashed edges is about 50 nm.

**Statistical analyses of the magnetic contrasts in the MFM images**

The MFM contrasts of the edges can be classified into two categories based on their contrasts. Type 1 edges show a bright contrast in the MFM images, with the magnetic moments oriented out-of-plane. Type 1 edges are marked by solid lines in the inset MFM image in Figure S3a. By contrast, Type 2 edges show no contrast in the images and are marked by dashed lines. Cross-sectional line profiles of eight Type 2 edges are shown in Figure S3a. Note that the line profiles are shifted to show the zero-signal platforms, which correspond to the Type 2 edges with in-plane magnetization. The averaged profile is shown in Figure S3b. These results unambiguously show that Type 2 edges with a width of approximately 50 nm do exist, although they are invisible in the images.


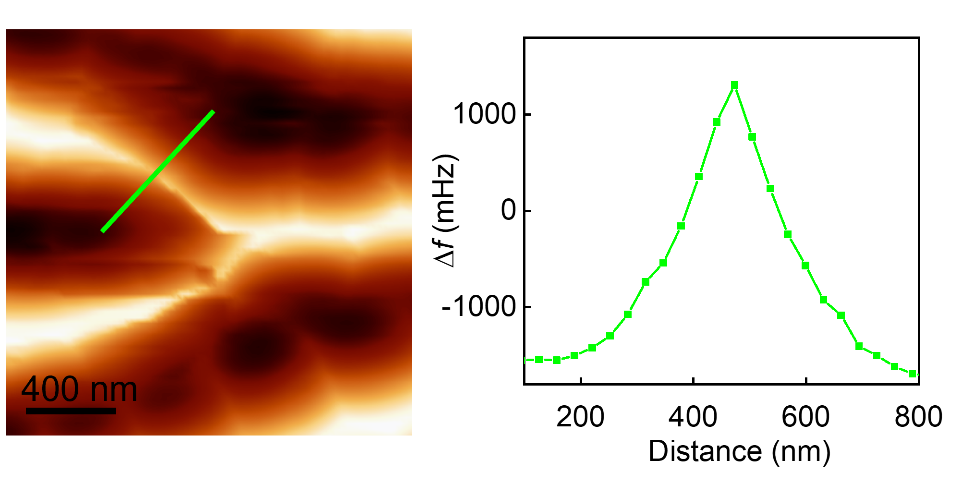


**Figure S4.** The flip of the magnetic moments in the transition region.

**
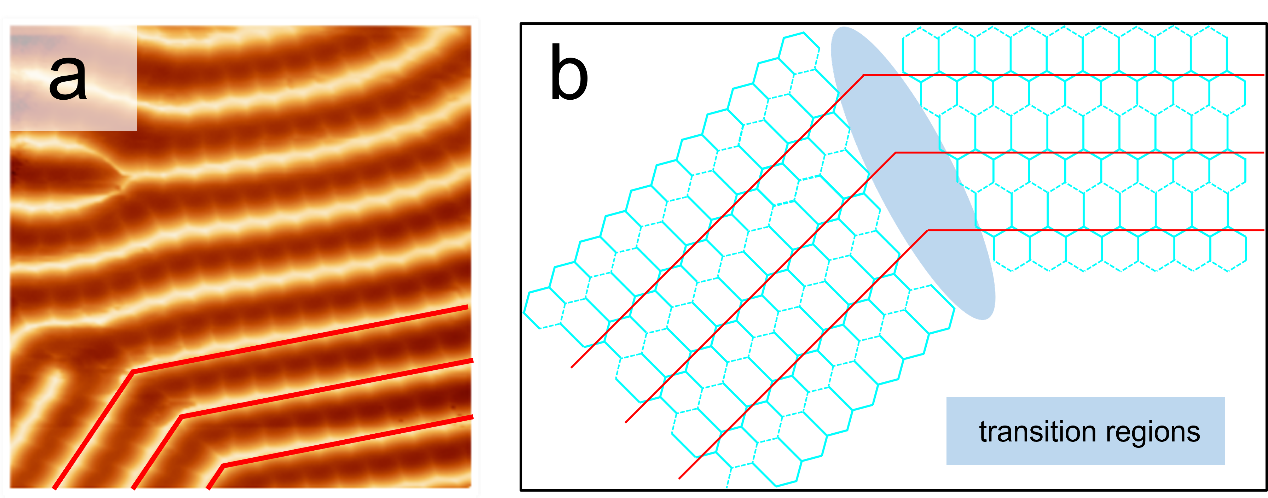
**

**Figure S5**. The orientation of the hexagons changes with the direction of the main lines (marked by solid red lines).


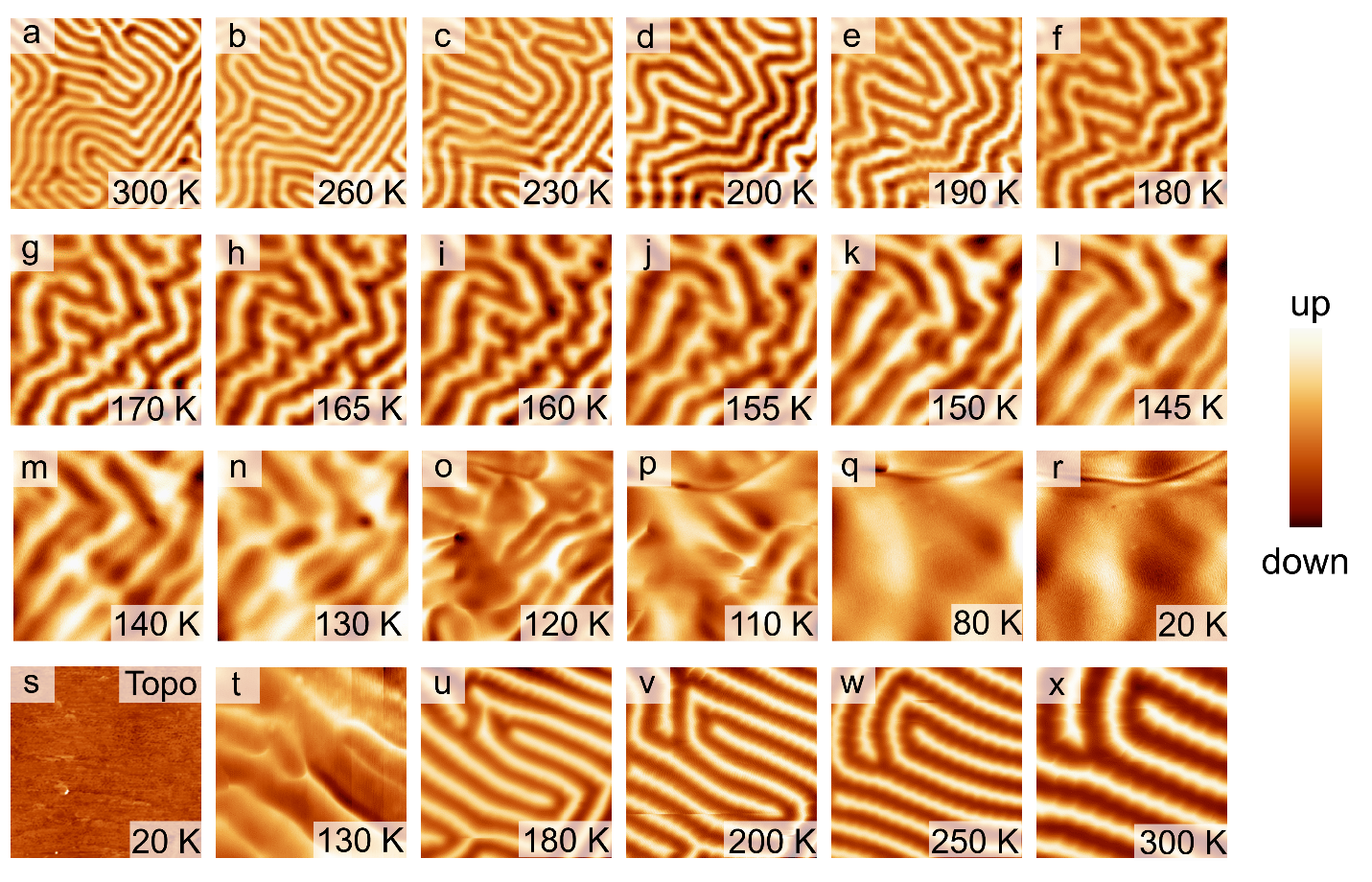


**Figure S6.** The full set of the MFM images taken during thermal cycling in zero field. All images are recorded at the same location. (**r**) and (**s**) are MFM image and corresponding topography image, respectively. No similar features are found at the position where the domain wall reside in the topography image. The colour scale for the topography image is 12 nm. The colour scales for MFM images are 7.12, 6.38, 6.07, 4.17, 3.33, 3.32, 2.02, 1.80, 1.59, 1.44, 1.19, 1.03, 0.87, 0.81, 0.70, 0.60, 0.57, 0.56, 1.87, 3.86, 5.89, 6.45, and 6.71 Hz, respectively. The scanned areas are 10.6×10.6, 10.5×10.5, 10.0×10.0, 9.2×9.2, 9.0×9.0, 8.7×8.7, 8.0×8.0, 8.0×8.0, 8.0×8.0, 8.0×8.0, 8.0×8.0, 8.0×8.0, 8.0×8.0, 8.0×8.0, 8.0×8.0, 8.0×8.0, 8.0×8.0, 8.0×8.0, 8.0×8.0, 7.0×7.0, 6.7×6.7, 6.2×6.2, 5.3×5.3 and 3.5×3.5 μm^2^, respectively.


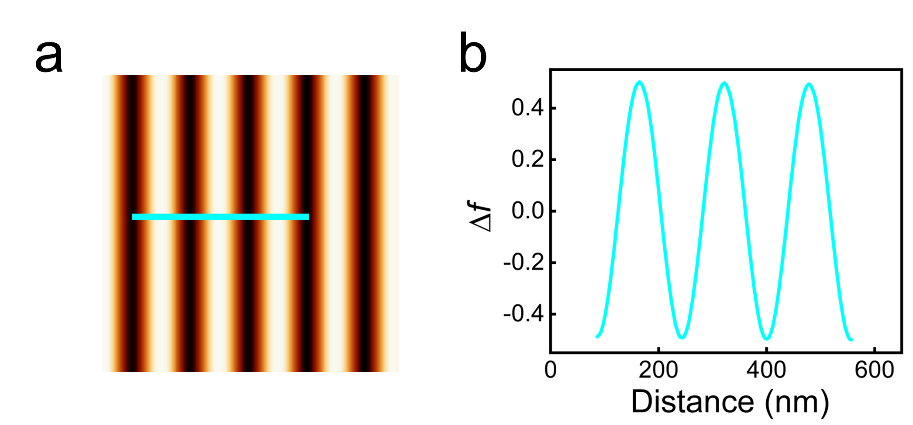


**Figure S7.** (a) Simulated MFM image of noncollinear stripes which are out-of-plane magnetized and (b) Line profile across these stripes. The magnetic state along the transverse direction is described by the sine function. Simulation was performed with the aid of MuMax3 software.


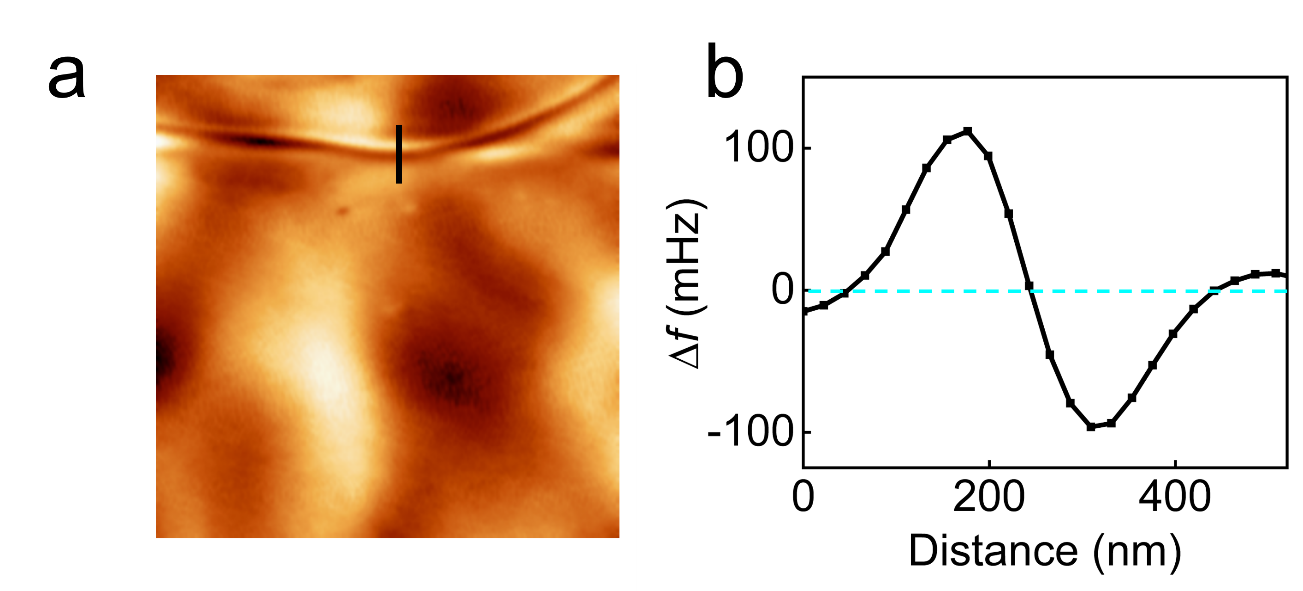


**Figure S8.** Line profile across the domain wall.


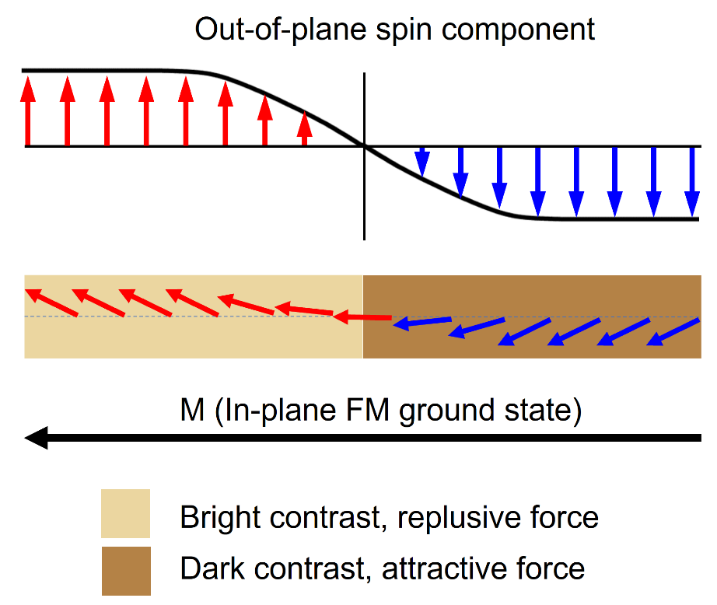


**Figure S9.** Schematic diagram of the in-plane FM ground state in a single domain. Upper panel shows the out-of-plane spin component, and lower panel shows the side view of the in-plane FM ground state.


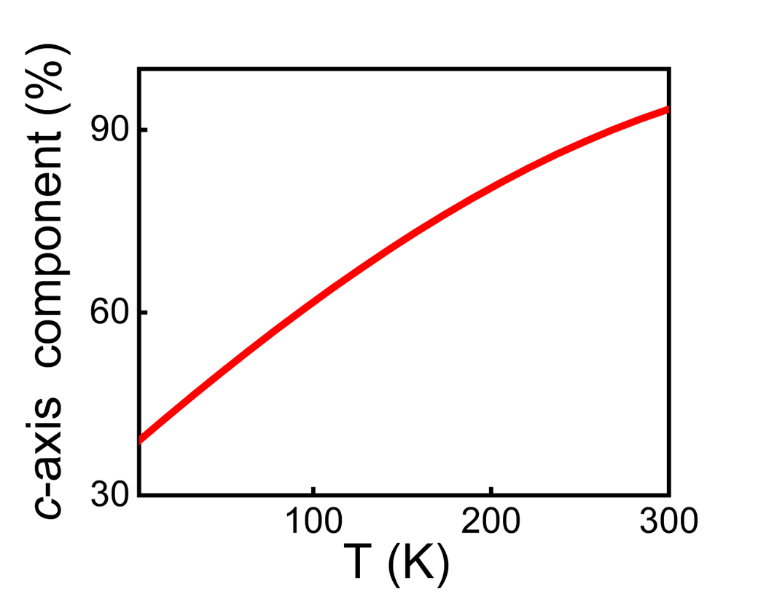


**Figure S10.** The variation in the portion of the moments along the *c*-axis with changing temperatures.

**Estimation of the portion of the moments along the *c*-axis**

First, the temperature dependence of the angle between M (magnetic easy axis) and the *c*-axis is estimated. As shown by the cyan line in Figure 1b, the linear increase in magnetization along the *ab*-plane indicates a spontaneous magnetization along the *ab*-plane as the temperature decreases. Considering that most moments lie in-plane at low temperatures, we set the magnetization value obtained at the lowest temperature (2 K) as the reference point. Then, the angle between the M and the *c*-axis can be estimated, as shown in Figure 1c. Second, the portion of the *c*-axis moment is estimated as:

$$Portion(\%)=100*cos$$

The key point in this method lies in the selection of the reference point. From our MFM data, we can find that the color scales (Δ*f* ) for MFM images taken at low temperatures are much lower compared to those taken at high temperatures. For example, the color scale for the image taken at 20 K is only 0.56 Hz, which is approximately 8% of the value at 300 K (Δ*f* = 7.12 Hz). Since the out-of-plane magnetization M*_c_*_-axis_ is proportional to | Δ *f* | (*Nat. Mater.* 2020, 19, 397-404), we can conclude that the magnetic moments are mostly aligned in-plane at low temperatures (especially at 2 K). Therefore, the selection of this reference point will not introduce significant errors in the estimation. This can be further confirmed by comparing it with the result reported in *Adv. Mater.* 2017, 29, 1701144, where the angles are calculated based on the temperature dependence of anisotropy constant. In Adv. Mater. 2017, 29, 1701144, the angle is reported to be 69° at 6 K and 22° at 300 K, whereas our method yields results of 66.4° at 6 K and 20.9° at 300 K. The comparison between these two methods confirms the rationale behind the selection of the reference point and supports the reliability of our approach.


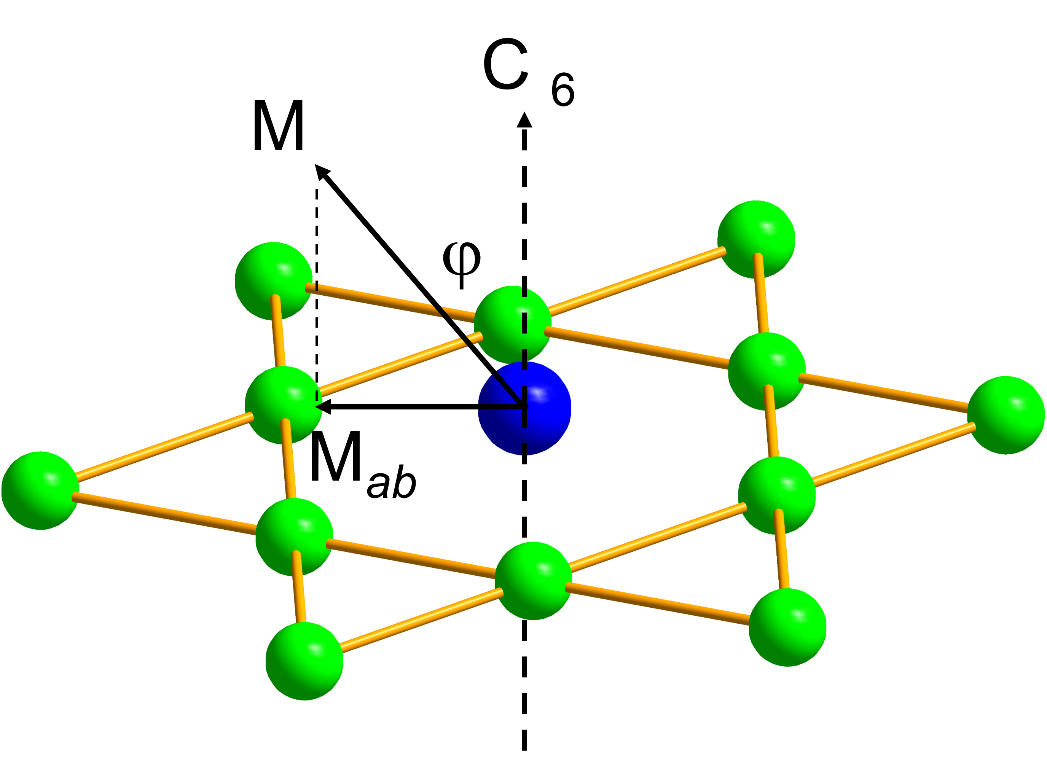


**Figure S11.** The schematic illustrates that the angle (ϕ) between the magnetic easy axis and the *c*-axis has the potential to disrupt the six-fold symmetry axis in magnetic textures.


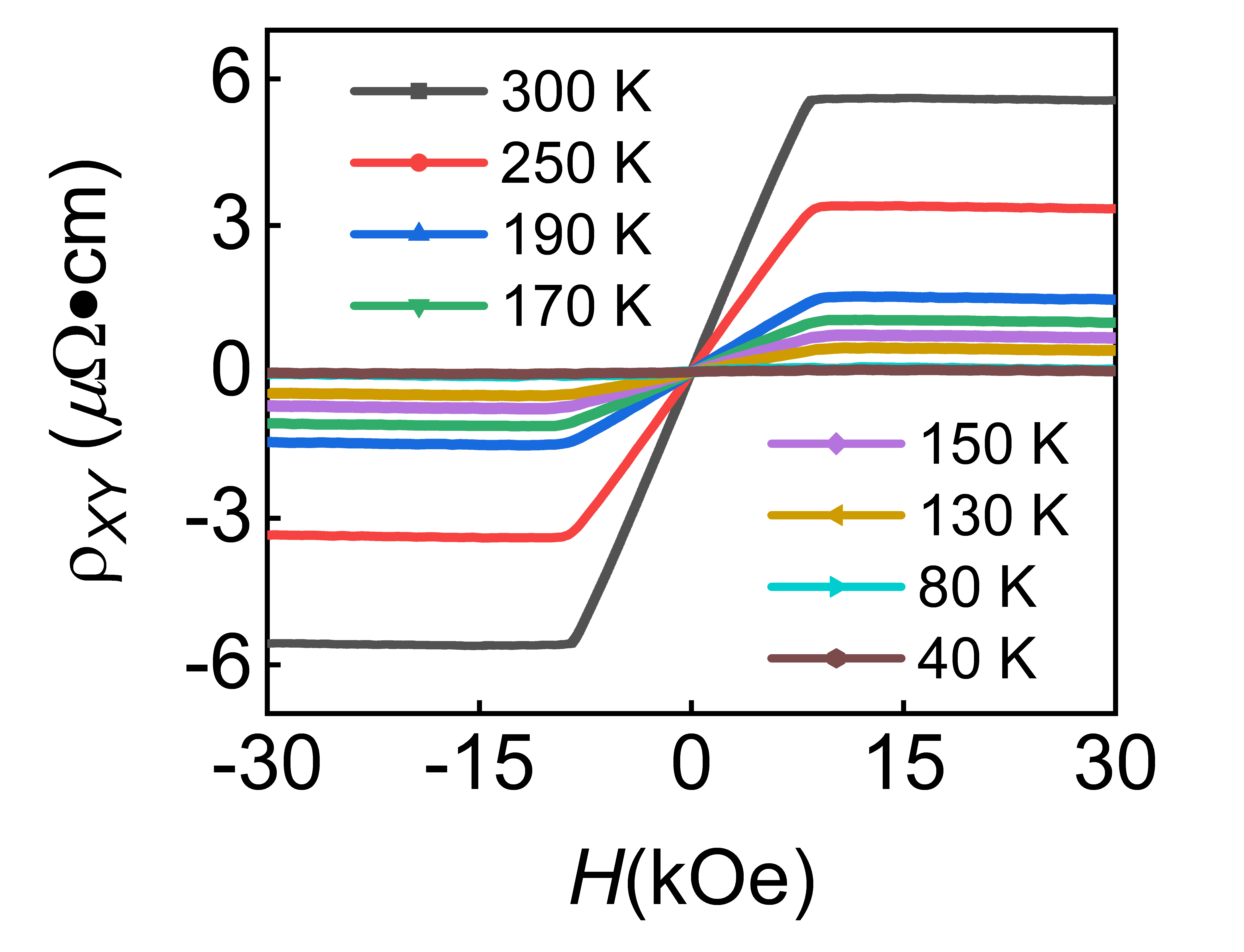


**Figure S12.** The transverse Hall resistivity (ρ_xy_) versus *H* at various temperatures for *c*-axis fields.
